# Accuracy and impact of spatial aids based upon satellite enumeration to improve indoor residual spraying spatial coverage

**DOI:** 10.1186/s12936-018-2236-2

**Published:** 2018-02-23

**Authors:** Daniel J. Bridges, Derek Pollard, Anna M. Winters, Benjamin Winters, Chadwick Sikaala, Silvia Renn, David A. Larsen

**Affiliations:** 1 Akros, 5 Reedbuck Road, Lusaka, Zambia; 2 University of Montana School of Public and Community Health Sciences, Missoula, MT USA; 3 Government of Zambia, Ministry of Health National Malaria Elimination Center, Lusaka, Zambia; 4 Syracuse University Department of Public Health, Food Studies and Nutrition, Syracuse, NY USA

**Keywords:** Malaria, Zambia, Indoor residual spraying, Electronic data capture, Spray effectiveness, Spatial coverage, mSpray

## Abstract

**Background:**

Indoor residual spraying (IRS) is a key tool in the fight to control, eliminate and ultimately eradicate malaria. IRS protection is based on a communal effect such that an individual’s protection primarily relies on the community-level coverage of IRS with limited protection being provided by household-level coverage. To ensure a communal effect is achieved through IRS, achieving high and uniform community-level coverage should be the ultimate priority of an IRS campaign. Ensuring high community-level coverage of IRS in malaria-endemic areas is challenging given the lack of information available about both the location and number of households needing IRS in any given area. A process termed ‘mSpray’ has been developed and implemented and involves use of satellite imagery for enumeration for planning IRS and a mobile application to guide IRS implementation. This study assessed (1) the accuracy of the satellite enumeration and (2) how various degrees of spatial aid provided through the mSpray process affected community-level IRS coverage during the 2015 spray campaign in Zambia.

**Methods:**

A 2-stage sampling process was applied to assess accuracy of satellite enumeration to determine number and location of sprayable structures. Results indicated an overall sensitivity of 94% for satellite enumeration compared to finding structures on the ground.

**Results:**

After adjusting for structure size, roof, and wall type, households in Nchelenge District where all types of satellite-based spatial aids (paper-based maps plus use of the mobile mSpray application) were used were more likely to have received IRS than Kasama district where maps used were not based on satellite enumeration. The probability of a household being sprayed in Nchelenge district where tablet-based maps were used, did not differ statistically from that of a household in Samfya District, where detailed paper-based spatial aids based on satellite enumeration were provided.

**Conclusion:**

IRS coverage from the 2015 spray season benefited from the use of spatial aids based upon satellite enumeration. These spatial aids can guide costly IRS planning and implementation leading to attainment of higher spatial coverage, and likely improve disease impact.

## Background

Indoor residual spraying (IRS) is a key tool in the fight to control, eliminate and ultimately eradicate malaria [1]. IRS was a primary vector control intervention employed in the Global Malaria Eradication Programme in the 1950s and 1960s. While the programme was far from a complete success, it did manage to eliminate malaria from a number of countries [2]. IRS reduces malaria transmission by killing endophilic mosquitoes that rest on walls following blood meals. Because IRS does not prevent host-seeking mosquitoes from taking a blood meal, an individual’s protection largely relies on the community-level coverage of IRS rather than household-level coverage. IRS is intended to reduce the age of the vector population and thereby reduce the potential to complete the cycle of transmission. Ensuring high and uniform community-level coverage should be the ultimate priority of an IRS campaign—low coverage IRS may offer no protection against malaria [3]. Implementation of IRS in malaria-endemic areas can be complicated by a lack of information available about both the location and number of households needing IRS in any given area. The last 50 years have seen numerous and significant advances in computing, communications, and mapping in disease control that could ease these complications in IRS implementation.

Spatial aids, i.e., maps, have been used in malaria control throughout history with perhaps the best example being the systematic elimination of *Anopheles gambiae* from Brazil in the 1930s [4]. Today traditional IRS campaigns vary on their use of spatial aids, and certainly vary in the quality of spatial data available. Spatial data available for IRS programmes in lower-income countries with high malaria burdens are highly limited, and typically include geospatial boundaries of districts, provinces, or regions and not actual locations of where people live. The lack of available data is particularly problematic for both the implementation and the evaluation of indoor residual spray programme. In implementation these challenges lie in actually spraying houses that are targeted for spray, and in evaluation these challenges lie in calculating and mapping coverage and monitoring success. These problems are not specific to IRS for malaria control. For example, in northern Nigeria the polio eradication campaign found that the use of spatial aids could aid micro-planning, assist in monitoring, and improve vaccination coverage [5]. Coupling the maps derived from satellite imagery with routinely monitoring vaccine teams with geographic information systems improved geographic coverage of polio vaccine by an estimated 40% in Kano state, Nigeria, which correlated with the disappearance of wild polio virus type 1 from the area [6, 7].

mSpray was developed to take advantage of advances in satellite imagery, cellular access, and other information technologies to improve IRS planning, delivery and monitoring by providing a fit for purpose spatial solution. The mSpray tool was developed and refined over multiple spray seasons namely in Zambia, but also in Madagascar, Zimbabwe and Namibia through field experience and user feedback. There have been a number of tool iterations, starting from its genesis as a basic spatial data collection tool on personal digital assistants in 2011. Since then, mSpray has increased in comprehensiveness, automation and resolution to the full spatial mapping, planning and field management tool it is today.

**Fig. 1:**
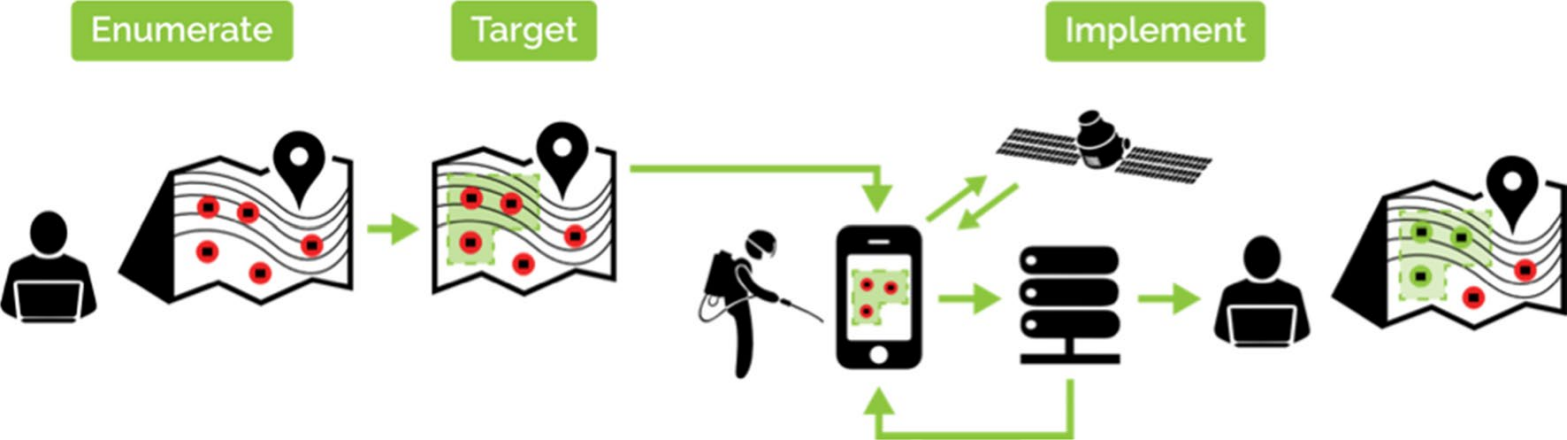
mSpray consists of enumeration of all structures from satellite maps, selection of households for IRS using a targeting methodology and finally spatial data collection with near real-time reporting/feedback. The figure shows from left to right, Enumerate—computer-based satellite enumeration, target—a map of enumerated structures (red dots) and target areas (green), implement—a tablet-based map to guide users to targeted structures where spatial data is collected, reported to a server and then visualised on intuitive interactive dashboards

mSpray encompasses three steps—enumeration, targeting, and spraying (Fig. 1). Enumeration consists of identifying all structures using publicly available satellite imagery [8], and only requires an internet connected laptop loaded with the open-source Quantum GIS program. Once enumerated, all structures, or a subset of structures can be targeted for IRS [9] and the progress of a spray campaign can be measured against this enumerated denominator. Finally, during the spraying itself, geo-located IRS data can be collected electronically offline, and with a working internet connection, automatically reported to a central server where spatial outcomes, e.g., spray coverage, are calculated and displayed through intuitive dashboards and maps to inform and drive daily decision-making.

While previous satellite enumeration exercises have been highly accurate [10, 11], the impact of spatial aids, such as paper-based maps, as well as an electronic data capture system such as mSpray on IRS coverage has not previously been demonstrated. This paper assesses the accuracy of satellite based enumeration in terms of its ability to identify sprayable structures and then measures the impact, in terms of improving spray coverage, that spatial aids based on the satellite enumeration had on IRS coverage following the 2015 IRS campaign in Zambia.

## Methods

### Study setting

The study was conducted in Nchelenge and Samfya districts in Luapula Province and Kasama district in Northern Province, Zambia (Fig. 1). Both of these provinces have above average national malaria prevalence in the under-five age group measured at 27.6 and 32.5% respectively in 2015 [12]. *Anopheles funestus* and *Anopheles gambiae* are the two main vectors in the area [13]. The population residing within these three districts practice traditional subsistence farming and is demographically similar.

### 2015 spray implementation

All three districts were enumerated using satellite imagery from freely available Google and Bing satellite imagery in 2014 for Nchelenge and Samfya or 2015 for Kasama. During the 2015 IRS operations conducted in the later months of 2015, the three districts had different levels of spatial aids supporting the IRS campaign. Nchelenge district employed the electronic mSpray data field collection tool which contained detailed spatial targeting maps based upon satellite enumeration pre-loaded on hand-held electronic tablet devices. Samfya district utilized paper-based targeting maps developed through the satellite-based enumeration method. Finally, Kasama received no spatial aids based upon satellite enumeration (Table 1). The differences in spatial aids provided to the district were determined by the implementing partners and not by the researchers.

**Table 1:** Three sample domains

District	Satellite enumeration date	Paper-based maps^a^	mSpray field implementation^b^
Nchelenge^a, b^	2014	Yes	Yes
Samfya^a^	2014	Yes	No
Kasama	2015	No	No

Spatial aid indicated by ^a^ representing use of paper*-*based maps during implementation or by ^b^ representing mSpray field implementation

The satellite enumeration and IRS targeting processes have been described in detail elsewhere [8, 9]. In brief, structures visible on satellite imagery are traced by trained enumerators using Quantum GIS. Enumerated structures are then grouped into spray areas, where houses within 250 m of each other grouped together into the same spray area. Spray areas are then prioritized based upon malaria incidence at the nearest health facility. Once prioritized, spray areas are identified through objective measures, districts provide input into which areas may be inaccessible during campaigns and which areas need to be sprayed based on local knowledge of malaria transmission.

### Data collection

**Fig. 2:**
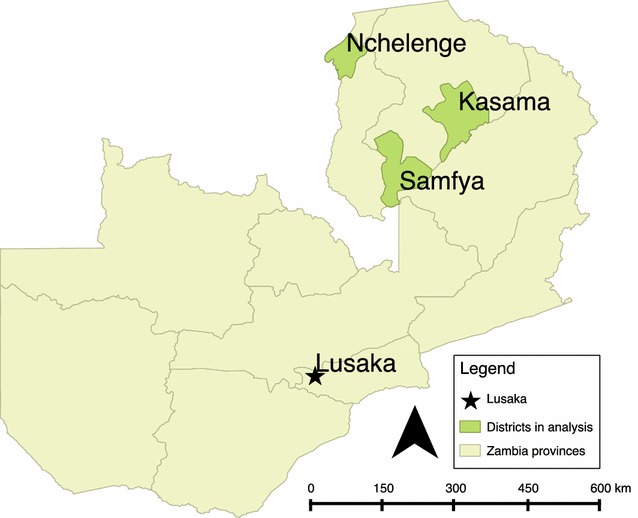
Map of the three study sites

To assess the accuracy of satellite enumeration to identify the number of sprayable structures within a spray area, a 2-stage sampling process was designed. Firstly, a sampling frame of all spray areas within the district was created and 30 spray areas for each district were selected probability proportionate to size. Secondly, teams of trained surveyors then canvassed random transects in each sampled spray area to map every existing sprayable structure found on the ground within each transect and conduct household interviews to assess IRS coverage (Fig. 2). IRS coverage was assessed in two ways, the presence of an IRS card showing the house had been sprayed in the campaign and through respondent self-report. In addition, surveyors noted the size of the house (average, smaller than average, or larger than average), and the type of roof (thatched or corrugated metal). Surveys were conducted in February 2016, approximately 4 months following the implementation of IRS in the study districts.

### Assessment of satellite enumeration accuracy

Geocoordinates of households located within transects during ground exercises were loaded into Quantum GIS version 2.0.1. Using the convex hull tool in Quantum GIS, which creates a polygon by tracing the outermost points in a group of points, polygons were created around households found during ground exercises for each transect and then the number of structures identified during satellite enumeration calculated within each transect’s convex hull. The difference between structures found on the ground and structures found during satellite enumeration provides an estimate of error for the satellite enumeration accuracy. Due to the inherent measurement error of GPS units, the creation of convex hulls was repeated but using a 10-m buffer around households found during ground exercises. Using the aggregate numbers of houses found during satellite enumeration and those found during ground sampling, a measure of sensitivity was defined as total houses enumerated by satellite divided by total houses in the area. This measure was repeated per sampled spray area. The data collection process did not generate “negative” households, or point locations without households. To determine a measure of overestimation of satellite enumeration the number of houses enumerated by satellite but not found on the ground is reported as a false negative.

### Assessment of spatial aids based upon satellite enumeration in improving IRS coverage

A post-only cross-sectional study of households in areas targeted for IRS was used to determine whether the deployment of spatial aids based on satellite enumeration was associated with the probability of whether a house, located in an area targeted for IRS, actually received IRS. As stated previously, each district received differing levels of mSpray deployment: Kasama District received a satellite enumeration only but no detailed maps based on the enumeration, Samfya district received detailed paper maps based on the satellite enumeration, and Nchelenge district received household enumeration and tablet maps based on satellite enumeration that were used in the field during the IRS campaign (Table 1). A logistic regression was used to determine if the probability of a house receiving IRS differed by district and therefore level of spatial aid, after adjusting for household size and construction type. Standard errors were adjusted for random transect. All analyses were conducted in Stata version 13.1.

## Results

### Accuracy of satellite enumeration

**Fig. 3:**
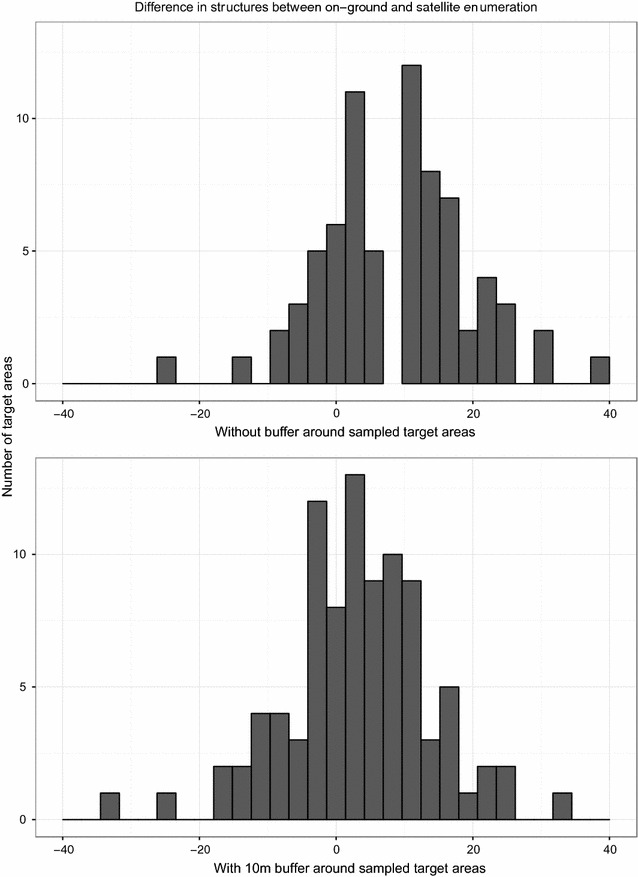
Difference in number of structures identified by satellite enumeration or on the ground in sampled target areas

During canvassing, a total of 4633 structures were identified along 92 separate transects in 60 target areas in the three districts during the ground canvassing. Of these, 941 did not contain a sleeping space and were thus classified as non-sprayable, leaving 3692 sprayable structures found on the ground. Across all transects, the number of structures found during ground canvassing ranged from 26 to 83, with a median of 37. Satellite enumeration classified 3449 sprayable structures in the same areas, the number of structures identified via satellite ranging from 8 to 77 with a median of 34. As a whole, satellite enumeration underestimated the number of structures in the sampled areas. By spray area, satellite enumeration slightly overestimated the number of sprayable structures, with the error number of structures per transect being normally distributed and centered at 2.4 when using the 10 m buffer to estimate number of structures found via satellite (Fig. 3). There was no significant correlation between the error in satellite enumeration and the number of structures in the transect (Pearson’s correlation coefficient = 0.2119). Throughout the entire area satellite enumeration had an estimated sensitivity of 94%. Per spray area, sensitivity ranged from 55 to 100%, with a median of 100%. The number of false negatives (houses not identified during satellite enumeration) in the sampled areas ranged from 0 to 25, with a median of 3.

### Interview acceptance

A total of 1013 households did not have an individual available for interview, leaving a sample size of 2679 to be interviewed. Nchelenge District had the lowest rate of interview acceptance and availability, with 62.7% of households identified available and willing to be interviewed. Kasama District and Samfya District had 80.6 and 78.4% of households identified available and willing to be interviewed, respectively.

### IRS coverage

IRS coverage in all three districts was well below the 85% threshold suggested by the WHO regardless of whether IRS was measured by interview or IRS card (Table 2). After adjusting for structure size, roof and wall type, households in Nchelenge District where the mobile application of mSpray was used during the IRS campaign were more likely to have received IRS than Kasama district, which had no maps based on satellite enumeration (adjusted odds ratio [AOR] = 1.8, 95% confidence interval [CI] = 1.1–2.9) (Table 3). The probability of a targeted structure being sprayed in Samfya district, which was provided with detailed paper-based maps, was also higher than in Kasama district but this difference was not statistically significant at the 95% level (AOR = 1.5, 95% CI = 0.9–2.7). The probability of a household being sprayed in Nchelenge district did not differ statistically from that of a household in Samfya District where mSpray was not used during field operations but the district was provided with detailed paper maps based on satellite enumeration (AOR = 1.2, 95% confidence interval = 0.7–1.9).

**Table 2:** IRS coverage across sampled districts

District	IRS coverage as measured by interview (95% confidence interval), n = 2679 households; 87 transects	IRS coverage as measured by card (95% confidence interval), n = 2679; 87 target areas
Kasama	54.5% (44.9–64.1%)	36.0% (26.7–45.2%)
Nchelenge^a, b^	68.6% (62.9–74.2%)	66.1% (58.9–73.2%)
Samfya^a^	64.9% (55.7–74.0%)	64.6% (55.8–73.4%)

Kasama, Nchelenge and Samfya all received different levels of spatial aid during IRS implementation (see Table 1). Spatial aid indicated by ^a^ representing use of paper-based maps during implementation or by ^b^ representing mSpray field implementation

**Table 3:** Factors associated with household being sprayed as verbally reported during interview

Covariate	Factor	Unadjusted OR (95% CI)	P value	Adjusted OR (95% CI)	P value
District	Kasama	Reference	Reference	Reference	Reference
	Nchelenge^a, b^	1.820 (1.140–2.906)	0.013	1.755 (1.072–2.873)	0.026
	Samfya^a^	1.539 (0.882–2.688)	0.128	1.479 (0.847–2.581)	0.166
Size of structure	Average	Reference	Reference	Reference	Reference
	Larger than average	0.758 (0.533–1.077)	0.121	0.991 (0.686–1.432)	0.962
	Smaller than average	0.692 (0.468–1.024)	0.065	0.723 (0.493–1.060)	0.096
Type of roof	Thatch	Reference	Reference	Reference	Reference
	Corrugated metal	0.690 (0.495–0.962)	0.029	0.993 (0.658–1.498)	0.972
Type of wall	Rough	Reference	Reference	Reference	Reference
	Smooth	0.657 (0.439–0.983)	0.041	0.693 (0.431–1.116)	0.130

Kasama, Nchelenge and Samfya all received different levels of spatial aid during IRS implementation, with Kasama having enumeration only; Samfya having paper-based maps developed from satellite imagery; and Nchelenge having the same type of paper-based maps as Samfya with the addition of in-field usage of the mSpray mobile application during spray implementation. Spatial aid indicated by ^a^ representing use of paper-based maps during implementation or by ^b^ representing mSpray field implementation

N = 2679 structures; 87 transects

## Discussion

IRS remains an invaluable tool in malaria control and elimination efforts, but operationally its deployment has not changed significantly from its inception. mSpray seeks to bring to bear new methodologies, devices and analyses to maximize the impact of IRS on malaria transmission.

The results from this study suggest that the use of detailed spatial aids improves community-level coverage of IRS campaigns. Furthermore this study corroborates those performed previously to demonstrate that accurate maps can be created by using publicly available satellite imagery and open-sourced GIS platforms [10, 11]. One challenge in implementing this study was the 4-month delay between the survey and the IRS campaign, due to logistical issues. While this could lead to recall bias or errors e.g. by interviewing households that moved after the campaign, it was uniform across the sampled areas and the analysis focused on differences between areas rather than on absolute values. Improvement in IRS implementation is greatly needed given the poor community-level coverage achieved by IRS programmes across sub-Saharan Africa [14]. These results suggest that improvement can be made by utilizing satellite-imagery based spatial aids during implementation.

To accurately calculate spray coverage of an IRS campaign, it is absolutely essential that the number of structures in an area is known. Traditional ground enumerations are laborious, expensive, and slow. In contrast, satellite enumeration has been estimated to be 22 times faster and 10 times less expensive than ground-based enumeration [8]. Satellite enumeration proved to be highly accurate in this study, with little error in identifying the number of sprayable structures in an area. This degree of accuracy suggests that satellite enumeration is not only a suitable replacement for ground enumeration, but that it is significantly more cost-effective and scalable [10, 11]. The satellite imagery used in this study was open-source and downloaded free from the internet. Combined with free tools like QGIS, minimal training needed for enumeration, and despite certain caveats e.g. extremely dense housing or forest can affect accuracy, there is little reason, not to use satellite enumeration to inform denominators for planning and monitoring an IRS campaign. Furthermore, this approach could be used to inform denominators for a host of other interventions such as ITN distribution, vaccination campaigns, or mass drug administration.

The use of mSpray during spray implementation was not found to be associated with improved IRS coverage above and beyond the use of detailed paper maps based upon satellite enumeration; however, this finding is limited by the low response rate in Nchelenge District. During implementation, the power of tablet-based maps lies in the ability to monitor coverage in real time, and any gains in coverage depend upon how supervisors utilize and respond to the real-time coverage data generated. More recent and forthcoming analyses from the 2016 and 2017 spray campaign in Zambia show that increased engagement of programme managers with electronic data delivered by mSpray led to improved IRS coverage. Although further research must be done, it is likely that this finding is due to the additional efforts and focus made in 2016 and 2017 to ensure programme managers are routinely accessing and using mSpray data during field implementation. Driving IRS decision making in this way will likely lead to increased spray coverage as managers are able to use real-time data to understand where lags in spray progress occur and address those quickly and accurately. Further, implementing any new technology takes time for users to accept and ‘buy into’ the system; for example in the Nigeria polio campaigns, the real gains in vaccine coverage were realized a few years after the first explorations with spatial aids and vaccine worker monitoring [7].

Another potential issue with the use of mSpray in the field lies in the IRS metric used by the implementing team. Unfortunately, IRS campaigns typically measure coverage using the number of houses found by the spray teams as a denominator. This metric does not reflect true spray coverage (proportion of target houses sprayed). Changing the metric of success for implementers would likely have a large effect on improving IRS coverage. Indeed, more accurately reporting true coverage may explain why some IRS campaigns see success in reducing malaria burdens whereas others do not.

While improvements need to continue to be made in the way that end-users interact with and understand IRS data, mSpray provides a robust tool to effectively manage IRS campaigns. The mSpray tool can capture data electronically, eliminating the need for paper-based forms and likely improving data quality while decreasing cost. For example, in Burkina Faso the move from paper-based data capture to electronic data capture in a demographic and health surveillance site led to 16% cost savings without affecting accuracy [15]; in Nepal, the use of tablet-based data capture during the demographic and health survey of 2011 improved data accuracy and availability [16]; and in South Sudan using tablets during polio vaccine campaigns improved the accuracy of data collected, particularly the geo-referenced data, and improved timeliness of data analysis [17]. Data collected electronically are easily available for subsequent spray season planning as well as evaluation of campaigns in real time. Refinement of insecticide procurements, targeting and spray plans will likely improve by utilizing mSpray data collected during the previous spray season. Finally, IRS data are available to programme managers quickly and online, therefore, providing near-real time feedback to funders, implementers and other stakeholders alike through the mSpray visualization tool.

## Conclusions

Spatial aids with accurate denominators are crucial in planning and monitoring success of public health interventions such as IRS. Satellite enumeration is accurate to estimate these denominators and detailed maps based on satellite enumeration improve intervention coverage.

## Abbreviations

IRS: indoor residual spraying
GIS: geographical information system
GPS: global position system
